# An ongoing case–control study to evaluate the NHS Bowel Cancer Screening Programme

**DOI:** 10.1186/1471-2407-14-945

**Published:** 2014-12-13

**Authors:** Nathalie J Massat, Peter D Sasieni, Dharmishta Parmar, Stephen W Duffy

**Affiliations:** Centre for Cancer Prevention, Wolfson Institute of Preventive Medicine, Queen Mary University of London, London, UK; Centre for Cancer Prevention, Wolfson Institute of Preventive Medicine, Barts’ and The London School of Medicine and Dentistry, Queen Mary University of London, London, UK

**Keywords:** Bowel cancer, Screening, Case–control, Incidence, Advanced stage

## Abstract

**Background:**

Colorectal cancer is the third most common cause of cancer death in both males and females in England. A national bowel cancer screening programme was rolled out in England between 2006 and 2010. In the post-randomised controlled trials epoch, assessment of the impact of the programme using observational studies is needed.

This study protocol was set up at the request of the UK Policy Research Unit in Cancer Awareness, Screening and Early Diagnosis to evaluate the effect of the current bowel cancer screening programme on incidence of advanced primary colorectal cancer.

**Methods/Design:**

All incident cases of primary colorectal cancer in England will be included. Cases will be matched to controls with respect to sex, age, area of registration and year of first invitation to screening. Each evaluation round will cover a 2-year period, starting from January 2012, and ongoing thereafter. In the first instance, a pilot will be carried out in a single region. Variables related to colorectal tumour pathology will be obtained to enable selection and matching of cases and controls, and to allow analyses stratification by anatomical subsite within the bowel. Cases at Duke’s stage B or worse will be considered as "advanced stage". The influence of sex will also be investigated. The incidence ratio observed in randomised controlled trials between controls (not invited) and non-attender invitees will be used to correct for self-selection bias overall. Screening participation at other national screening programmes (cervical, breast) will also be collected to derive a more contemporaneous adjustment factor for self-selection bias and assess consistency in self-selection correction in female patients.

Full ethical approval was obtained from the Health Research Authority.

**Discussion:**

The case–control design is potentially prone to a number of biases. The size of the planned study, the design specifications and the development of analytical strategies to cope with bias should enable us to obtain accurate estimates of reduction in incidence of advanced stage disease. The results of analyses by sex and anatomical subsite may highlight the potential need for sex-specific recommendations in the programme.

## Background

In England, colorectal cancer (CRC) is the third most common cancer in men after prostate and lung cancer and in women, after breast and lung cancer [1].

Meta-analyses of the randomised controlled trials (RCTs) have demonstrated the efficacy of biennial screening with guaiac faecal occult blood test (gFOBt) for haemoglobin in reducing *mortality* from CRC (rate ratio = 0.88, 95% CI 0.83-0.94, reviewed in [2]), while generally no significant effect of screening on *incidence* was found after 14 to 20 years of follow-up. Only one trial, performed in the USA, demonstrated any significant reduction in CRC incidence, but the validity of this result may be limited due to the fact that an unusually large proportion of subjects in the intervention arm underwent colonoscopy [3]. However, in all the trials, the reduction in mortality was accompanied by a reduction in advanced stage disease [4–7].

The English Bowel Cancer Screening Programme (NHS BCSP) began in July 2006 and rolled out incrementally across the country, achieving national coverage in 2010. It initially offered screening to all men and women between the ages of 60 and 69 years, resident in England and registered with a general practitioner. In 2010, it was extended to include everyone up to the age of 74 years inclusive. Individuals aged 75 and over may also self-refer into the programme [8, 9].

A major issue to be addressed by the UK Policy Research Unit in Cancer Awareness, Screening and Early Diagnosis (PRU), is the evaluation of the effects of the policy of CRC screening as delivered by the current national programme in terms of anticipated benefits. This issue is particularly relevant as the uptake of population-based gFOBt screening in the UK is low compared with, for example, that of mammographic screening [10], and also varies by age, sex, ethnicity and deprivation status [11–13].

In the post-RCT epoch, assessing the impact of the NHS BCSP requires the use of observational studies. Recently, Libby and collaborators [14] reported a significant 10% reduction in CRC mortality among invited individuals invited for screening as part of the UK CRC screening pilot studies in Scotland (2000–2007, three biennial rounds of invitation) when compared to a cohort of uninvited controls matched for age, gender and deprivation, and followed up to 31 December 2009.

In an ecological study, the same group found trends towards earlier stage and less advanced disease at diagnosis as well as decreasing mortality rates among individuals invited for screening as part of the pilot compared to general population individuals not yet invited for screening [15]. However, results from trends studies have limited validity [16].

We chose to address the policy questions using a retrospective matched case–control design because it is a powerful tool which has previously been used to great effect in the assessment of the NHS Cervical Screening Programme (NHS CSP), informing policy on screening intervals and age ranges [17], and recently, in the assessment of the NHS BSP [18].

Because the NHS BCSP is relatively recent (2006 onwards), long-term goals on mortality reduction may currently be difficult to assess using a retrospective design; however, this type of design will be helpful in investigating changes in incidence of advanced stage CRC, which in turn will indicate the likely future effect on mortality from the disease.

In the UK, CRC mortality has been declining in the population since the 1970s: over the last decade death rates have dropped by around 14%; in contrast, incidence rates, in particular in men, have been increasing over that same period [19, 20], making the question of the impact of the NHS BCSP on incidence of advanced stage primary CRC a very important one.

In addition, the male-to-female ratio for CRC incidence rates varies for different parts of the large bowel [21]: the majority of rectal cancer cases occur in men (63%), while colon cancer cases are approximately evenly divided between men (53%) and women (47%), suggesting the sensitivity of a particular screening strategy may differ between sexes [2].

To our knowledge, no case–control studies have been published which looked at the effect of gFOBt on CRC incidence. Two studies have been published which assessed the impact of immunochemical faecal blood test (FIT) screening on CRC incidence in Japan, in separate settings [22, 23] but only one of them investigated the effect of FIT on incidence of advanced stage disease [22]. In this study, cases (N = 349) diagnosed with advanced stage CRC (T2-T4 in TNM classification) aged 40 or over were compared to general population controls (3 per case) matched for sex, age and area of residence, with respect to screening history prior to diagnosis. The authors found a significant reduction in the incidence of advanced stage CRC in those who attended screening within 3 years prior to the date of diagnosis (OR = 0.54, 95% CI 0.30 – 0.99). No adjustments were made.

In view of the above, there is a need for a large case–control evaluation of the effect of the NHS BCSP on incidence of advanced stage CRC with appropriate methodology for coping with bias, notably self-selection bias. Meeting the above aims will entail retrieving information on tumour pathology variables, and on screening participation at other national screening programmes. This will be an ongoing biennial evaluation to ensure that the programme continues to deliver the anticipated health benefits, and to potentially improve the programme by identifying good and bad practices.

The case–control design is potentially prone to a number of biases, in particular some that could confer a bias *in favour* of screening; those will be addressed either at the design, or at the analysis stage of the study.

## Methods/Design

### Study design & objectives

The study will be a matched comparison of recently diagnosed/incident advanced stage primary CRC (colon, rectosigmoid junction and rectum) cases with general population controls free of advanced stage disease, with respect to screening exposure strictly prior to the case's date of *index/first diagnosis* (set as pseudo-diagnosis date in controls). There will be two controls per case.

The primary objective will be to determine whether the event of having been screened prior to diagnosis is superior to not having been screened in terms of incidence of advanced stage primary CRC. In addition, we plan to assess the benefit by time since last negative screen and number of screens among other measures of exposure. The secondary objective will be to consider the effect of screening on overall CRC incidence, regardless of stage.

A methodological objective of this protocol is to apply a procedure using data from RCTs and from other cancer screening programmes, in this case, from the cervical (NHS CSP) and the breast (NHS BSP) screening programmes, to adjust the analysis of the case–control studies for self-selection bias.

### Setting & source population

The background, implementation and organisation of the NHS BCSP are described in detail elsewhere [12]. For this study, all individuals who were invited to participate in the NHS BCSP in England (2006 onwards), and did not express dissent to their records being used for research, will be targeted.

The evaluation will begin with a pilot phase in one region in England using cases that were diagnosed with CRC between the 1st January 2012 and the 31st December 2013. The initial main phase of the study will then be undertaken, which will cover the whole of England or, those regions (i.e. previous cancer registries) for which tumour pathology variables will be available from 1st of January 2014. In achieving this, we shall put mechanisms in place so that the exercise can be carried out on a biennial basis.

### Participants: selection of cases & controls

#### Population of incident cases

All individuals who have had primary CRC diagnosed aged 60–89 within the specified 2-year study period will be selected. The *index diagnosis* will be either a first or a subsequent primary CRC and, therefore, individuals may have had a previous history of primary CRA or CRC (Duke A only) prior to the date of their *index diagnosis*. Cases may have died from primary CRC (as stated in part 1 of the death certificate) during the specified 2-year study period, or afterwards. DCO cases will be excluded. For subsequent phases of this evaluation (main phase onwards), individuals will not have been included as cases in previous evaluation periods, although they may have been included as controls.

#### Population of case-matched general population controls

For each case, two individuals of the same sex as the case will be selected from the general population who were registered in the same National Health Applications and Infrastructure Services (NHAIS) system as the case at the case's date of *index diagnosis*, were alive at the case’s date of *index diagnosis*, were born within 1 month either side of the case’s date of birth, and had had their first invitation to bowel screening in the same year as the case. If no matched control is found for a particular case with a date of birth within 1 month either side of the case's date of birth, the range will be expanded to 3 months either side (or 6 months either side if the case’s age at *index diagnosis* of primary CRC is over 75). For subsequent phases of this evaluation (main phase onwards), they will not have been included as population controls in previous evaluation periods.

Incident advanced stage cases and matched general population controls will be selected a posteriori by the PRU according to various designs as described in Table 1. In the initial stages of the evaluation, incident cases will be restricted to those individuals with Duke’s stage B or worse primary CRC at *index diagnosis* (Designs 1–3). Data permitting, we will also include individuals diagnosed with a Duke's stage A primary CRC during the study window but who were subsequently found to develop metastases (i.e. the cancer becomes stage B or worse) in order to avoid introducing a bias *in favour* of screening by excluding them from the population of cases and underestimating the proportion of Duke B+ tumours among screen-detected. Indeed, metastases are more likely to be early and therefore minimal and so missed in screen-detected compared to symptomatic cases; as a consequence, a minority of screen-detected tumours may be classified as having a less advanced stage leading to the underestimation of screening exposure in cases, and creating a bias *in favour* of screening.

**Table 1:** **Proposed case–control designs and analyses**

Study	Criterion	Cases	Controls
**Design 1**			
	Previous diagnosis	None	None
		CRA	CRA
		Duke A CRC	Duke A CRC
	Index diagnosis	Duke B+ CRC	_
	Analysis	Exposure to screening prior to *first diagnosis* of case	
		Controlling for previous history of CRA & CRC	
		Adjusting for screen-detection of CRC	
**Design 2**			
	Previous diagnosis	None	None
		CRA	CRA
		Duke A CRC	Duke A CRC
	Index diagnosis	Duke B+ CRC	_
	Analysis	Exposure to screening prior to *index diagnosis* of case	
		Controlling for previous history of CRA & CRC	
		Adjusting for screen-detection of CRC	
**Design 3**			
	Previous diagnosis	None	None
		CRA	CRA
	Index diagnosis	Duke B+ CRC	_
	Analysis	Exposure to screening prior to *index diagnosis* of case	
		Controlling for previous history of CRA	
**Design 4**			
	Previous diagnosis	None	None
		CRA	CRA
	Index diagnosis	Any CRC (Duke A-D)	_
	Analysis	Exposure to screening prior to *index diagnosis* of case	
		Controlling for previous history of CRA	
**Design 5**			
	Previous diagnosis	None	None
		CRA	CRA
	Index diagnosis	Any CRC (Duke A-D)	_
		Screen-detected	Last screened in same year as case index screen
	Analysis	Exposure to screening prior to *index diagnosis* of case	
		Controlling for previous history of CRA	

CRA: Colorectal Adenoma; CRC: Colorectal Cancer.

Duke A CRC: CRC diagnosed at stage Duke A; Duke B+ CRC: CRC diagnosed at stage Duke B, C or D.

Matched general population controls will be restricted to those individuals who have never had stage B or worse primary CRC, but may have had early stage primary CRC (Duke’s stage A) or CRA prior to and including the case's date of *index diagnosis*. The majority of controls, will, however, never have had CRC (or CRA). In later stages of the evaluation, all incident cases will be considered (Design 4).

All cases and controls will appear on the HSCIC database at Exeter and will have available data on bowel, cervical (for women aged 60–64) and breast (for women aged 60–74) screening strictly prior to the date of *index/first diagnosis* of the case, depending on the analysis (that is, they have been invited to screening at least once prior, see Table 1 for details). Bowel, cervical and breast screening history as well as primary CRC pathology variables will be retrieved for all cases and controls. In addition, history of previous CRA and corresponding tumour pathology variables will be obtained.

A detailed description of the subject selection criteria and matching algorithm will be created in collaboration with our NHS partners.

### Data sources & collection

Recently diagnosed cases will be obtained from the Northern and Yorkshire Cancer Registry and Information Service (NYCRIS) which is the lead registry for colorectal cancer. This cancer registry has linked the NHS BCSP data to the National Cancer Data Repository (NCDR) up until 2010 and is planning to update this linkage on a regular basis. Matching general population controls will be retrieved from the Health & Social Care Information Centre (HSCIC) database at Exeter. Tumour pathology variables will be obtained from the NCDR, including Dukes’ stage of disease at diagnosis which is the only currently nationally available staging variable. Detailed pathology information is also available via the NHS BCSP [8]. Bowel cancer, cervical and breast screening variables will be obtained from the HSCIC.

The data will be checked and cleaned by the PRU Senior Data Manager, transferred to separate Oracle tables, and stored on a UNIX server kept in a secure server room within the Wolfson Institute of Preventive Medicine. Access to the Oracle database is from PCs on the Queen Mary University of London secure network using SQLNET.

All data will be processed in accordance with NHS Information Governance guidelines (NHS IG Toolkit, https://www.igt.hscic.gov.uk/).

### Variables

A list of all the variables to be retrieved for all subjects, cases and controls (where applicable) during the ongoing evaluation study is presented in Table 2. Villousness or high-grade dysplasia, size (≥10 mm) and number of adenomas (≥3) have been found to be the most important predictors of future CRC [24, 25]. Anatomical subsite may also affect risk as sensitivity of investigation methods may differ between location (e.g. see [2] for differences with gFOBt).

**Table 2:** **Variables of interest for cases and controls**

Patient characteristics	Primary CRA & CRC pathology variables ^2^	Screening variables ^3^
Patient unique ID	Tumour unique ID	Screening Health Authority cipher (NHAIS)
NHS number	Tumour status	Screening office code
Sex	Tumour description	Screening office reference
Case or control	ICD-10 code	Year of first invitation to bowel cancer screening
	• C18 - Colon	
	• C19 - Rectosigmoid junction	
	• C20 - Rectum	
Date of birth	Dates of diagnoses (all CRAs & CRCs)	**For bowel/cervical/breast screening**
Patient status (Alive or dead at end of study period)^1^	Tumour detection mode (all CRAs & CRCs)	Episode number
Date of death (where applicable)	Anatomical subsite (all CRAs & CRCs)	Episode type
Age at death (where applicable)	CRC Dukes' stage (& any subsequent restaging)	Episode date
Date of *index tumour* diagnosis	CRA villousness	Screen date
Age at *index tumour* diagnosis	Tumour size (all CRAs & CRCs)	End code
Died of primary CRC (where applicable)	Treatment/procedure at diagnosis (all CRAs & CRCs)	Flag: NHS or Screening Programme registration by age 60
Cancer registry	Any further treatment/procedure (all CRAs & CRCs)	
Geographical area (as available in NCDR)	*Optional:* Dates of treatment (all CRAs & CRCs)	
IMD quintile (or postcode)		
Ethnicity		

^1^For cases and controls who died after date of *index diagnosis* of the case.

^2^Those variables will be retrieved for each neoplasia occurrence.

^3^All screening history.

### Power calculation

For the main phase of the study, all cases complying with the selection criteria in the whole of England covering the 2-year evaluation period will be collected. We anticipate biennial accrual of thousands of CRC cases (e.g. 33,218 - 18,590 male and 14,628 female cases - in 2010 [19]), for which statistical power will not be an issue and for which there will be considerable precision.

For the pilot phase of the study, the sample size was estimated to enable sufficient power to answer the primary objective with relative confidence.

Nakajima and collaborators [22] found that the odds ratio of developing advanced stage CRC for those screened within 2 years before the diagnosis versus those not screened using a FIT was 0.76 (95% CI 0.45 – 1.28) when both interval and screen-detected cancers were included in the analysis. We will therefore assume an odds ratio of 0.8 as FIT has been reported to have a higher sensitivity compared to gFOBt (reviewed in [2]).

We anticipate 64% agreement of exposure for matched cases and controls. We further anticipate 55% exposure for cases, 59% for controls. This would imply that for 39% of case–control pairs, both case and control would have been exposed to screening, for 25%, neither would have been exposed, for 16%, the case would have been exposed and not the control, and for 20% the control would have been exposed and not the case (illustrated in Table 3). This would give the expected odds ratio of 0.8. To have 90% power to detect this odds ratio as significant would require 259 discordant pairs and therefore 719 cases with one control per case [26]. As we propose to have two controls per case, 719 cases for the pilot study is considerably more than is strictly needed as the number of potential discordant pairs will be substantially increased.

**Table 3:** **Power calculation assumptions around exposure of cases and controls to bowel cancer screening (percentages)**

%	Cases Screened	Cases Not Screened	Total
Controls - Screened	39	20	59
Controls - Not screened	16	25	41
Total	55	45	100

Missing data for tumour pathology variables can be close to 30% with more advanced tumours more likely to have missing pathology data. In a recent retrospective observational study which used NCDR data, Dukes’ staging was available for 75% of the tumours registered between July 2006 and December 2008, however this percentage is likely to increase as time goes on (EJA Morris, personal communication).

Ninety percent of symptomatic CRC cases have been shown to be of Duke's stage B or worse [27], but a proportion of 80% at pathological stage B or worse would be a conservative estimate. Thus, only 56% (0.70 [missingness] × 0.80 [stage B+]) of the registered CRC cases will qualify for the study, and we will therefore inflate the sample size to select N cases so that 0.56*N = 719, i.e. around 1,300 cases.

As we require information regarding disease stage and other tumour attributes to select the cases a posteriori, we are planning to collect pilot data from at least 2,000 cases and twice that number of controls as a further failsafe measure. In addition to providing ample power for an odds ratio of 0.8 overall, this will also (1) provide tighter confidence intervals therefore impacting on significance, (2) confer the same power for an odds ratio of 0.7 in a subgroup comprising half the study population (for example in males, females or a particular subsite), and (3) allow for the investigation of the effect of different screening intervals. It is not clear what we should expect in subgroup analyses, but the observed results in the pilot will guide us in terms of the main study.

### Bias & effect modification

The case–control design is potentially prone to a number of biases [28], in particular some that could confer a bias *in favour* of screening, and which are addressed either at the design stage by choosing appropriate selection criteria, or at the analysis stage by using suitable statistical methods. The selection of participants and choice of analytic measures of exposure (see Participants: selection of cases & controls and Statistical Methods sections, respectively) are crucial to ensure accuracy.

#### Exposure opportunity (frequency) bias

Once diagnosed with cancer, the cases come under clinical management and do not continue with routine screening as before. The controls, however, will continue to attend screening. To avoid a potential bias *in favour* of screening, controls are given a pseudo-diagnosis date that is the same as that of their matched case and screening history is only considered up to that date [29].

In contrast, the fact of the case having necessarily a diagnosis of cancer and a control usually not having such a diagnosis, induces an artificially higher retrospective probability of screening exposure in the cases, a bias *against screening* (i.e. the screen-detected cases will always have this screen recorded at diagnosis, whereas the large majority of controls will not at pseudo-diagnosis date). Simply excluding the detection screens of the cases from the histories would bias the results *in favour* of screening. A driver of this bias is prevalence screening and, as the NHS BCSP is a relatively young programme with at most 3 rounds at the planned start of the pilot phase (January 2012), this bias may initially be present although it is likely to decrease for subsequent evaluations.

The extent of screening opportunity bias will be investigated first, by applying an analytical correction to the odds ratio using the method developed by Duffy and collaborators [30], and second, by performing sensitivity analyses in which the date of pseudo-diagnosis for controls whose matched cases have had a screen-detected *index diagnosis* will be extended by up to 3 years, the estimated average sojourn time for each screen-detected case, to counteract the artificially higher retrospective probability of screening exposure in cases [29]. A shorter sojourn time may however be more appropriate for screen-detected tumours of stage Duke B or worse [31].

In addition, we will perform a sensitivity analysis using screen-detected cases only. We will aim to establish whether such cases were screened less often in the past than matched controls who were screened at the time of detection screen of the case [32, 33]. In this analysis, controls that have had a screen in the same year as the *index screen* (detection screen) of their matched case will be included. The *index screen* will be excluded and the effect of prior attendance at screening on incidence of advanced stage primary CRC will be investigated. The impact of being invited to bowel cancer screening prior to *index screen* on CRC incidence may also be investigated.

But the major source of bias in case–control studies where controls are selected from the general population is potential self-selection bias [34].

#### Self-selection (volunteer) bias

Individuals who accept the invitation to screening (attenders) may have an a priori better health status compared with individuals who do not (non-attenders). In the case of bowel screening, gFOBt kit return has been shown to be significantly lower for postcode sectors with poor health [35]. Therefore attenders at screening may be less likely to acquire and potentially die from colorectal cancer. We would anticipate that this will confer a bias *in favour* of screening. Although this bias *in favour* of screening is unavoidable at the design stage, it can be approximately corrected for in the statistical analysis.

1. The regression analyses will be with adjusted for the Index of Multiple Deprivation (IMD), an area-based measure of relative deprivation, derived from residents’ postcodes based on census statistics for overcrowded housing and other factors, and believed to be the main confounding factor relating to both the exposure (i.e. the decision to attend screening), and the outcome.
2. The method developed by Duffy and collaborators [36] will be used *for all patients* to correct the estimated odds ratio using data on participation from the RCTs of CRC screening, in particular data from the Nottingham trial (Individual data obtained for the Nottingham trial from the authors JH Scholefield & SM Moss). In addition, data from regions with population characteristics (total population of screening age 60–69, deprivation, ethnicity, urbanisation status) comparable to areas offered gFOBt screening may be collected from the early stages of the NHS BCSP implementation (e.g. 2006–2007) when the programme had not been fully rolled out [9]. The following estimates may then be derived to potentially help adjust for self-selection bias: (i) CRC incidence among invited (region 1) screened individuals (i.e. risk among attenders), (ii) CRC incidence among invited (region 1) individuals who were not screened (i.e. risk in non-attenders), and (iii) CRC incidence among individuals who were not invited (and not screened, region 2).
3. The method developed by Duffy and collaborators [36] will also be used *for women only* to correct the estimated odds ratio using data on participation in the NHS CSP (cervical) and/or NHS BSP (breast) screening programmes, rather than data on participation from the RCTs of mammographic screening. In the absence of self-selection, the relative risk of primary CRC (death/incidence) associated with bowel cancer screening for non-attenders at cervical or breast screening would be expected to be equal to the relative risk in cervical or breast screening attenders (after adjusting for bowel cancer screening). In the Nottingham RCT, the incidence of primary CRC (all stage) was about 13% higher in non-attenders to bowel cancer screening compared to attenders (Individual data obtained for the Nottingham trial from the authors JH Scholefield & SM Moss). The crucial element in correcting for self-selection bias is the risk ratio for non-attenders versus attenders to breast/cervical screening. Thus the correction is estimated only from data on women as both other screening programmes apply to women only. This will be calculated for women who attended bowel screening at least once and for women who did not attend a single screen, to control for attendance at bowel cancer screening. A range of denominator values for the risk ratio will be assessed for sensitivity. One could compare primary CRC rates (i) in women who have had breast/cervical screening - but not bowel screening -with women who have had neither breast/cervical nor bowel screening; and (ii) in women who have had *both* breast/cervical and bowel screening with women who have only had bowel screening.

Data for women with age at diagnosis/pseudo-diagnosis between 60 and 69 year-old who would have been invited to both the bowel and breast screening programmes will be used. Alternatively, data for women with age at diagnosis/pseudo-diagnosis between 60 and 64 year-old who would have been invited to both the bowel and cervical screening programmes will be used. Also, in women under age 60, one could compare primary CRC rates in women who have, or have not had, breast/cervical screening, hence by-passing the positive confounding between uptake of the various screening programmes.

The national average participation rate at bowel cancer screening will be obtained for men and women from the NHS Annual Report on Bowel Cancer Screening [37, 12]. For the pilot phase, a single self-selection factor will be estimated as the data will cover one region only; for the national phase, regional factors may be estimated to assess variation in self-selection between regions.

Self-selection will be also addressed at an individual woman level by adjusting the regression model for participation in the other screening programmes, with careful adjustment for the confounding between attendances in different programmes.

### Statistical methods

Case–control study analyses will be conducted using conditional logistic regression. Matching factors (i.e. age, area and year of first invitation to bowel cancer screening) are controlled for in design, and additional analyses will be stratified by sex and anatomical subsite. All statistical analyses will be performed using the statistical software STATA version 12 and/or R version 2.13.0.

The ***primary objective*** of this case–control will be to assess the effect of various measures of participation in bowel cancer screening *strictly prior to* the case’s date of *index/first diagnosis* on incidence from advanced stage primary CRC (see details in Table 1). The primary measure of participation to screening will be whether an individual ever attended at least one screen episode prior to diagnosis. Secondary measures will be the total number of screens, the time since last screen, the time since penultimate screen, the interval between last screen and penultimate screen, the maximum interval between 2 screens, the average interval between 2 screens, the total number of invitations, the patient's age at first screen, and the patient’s age at last screen.

‘Time since last screen’ will give estimates of the likely benefit of screening at different intervals. It will include whether that time span fell within the past two years, as this corresponds to the NHS BCSP interval and approximates the estimated preclinical screen-detectable period (PCDP).

***The secondary objective*** will be to consider the effect of screening on overall CRC incidence, regardless of stage. Similar measures of participation will be used.

*Self-selection* and *exposure opportunity* biases will be addressed using the methods described in the *Bias & Effect modification* section.

Secondary analyses will consider time since last screen stratified by age at diagnosis/pseudo-diagnosis, by *index/first tumour* detection mode, and by region (Main phase only). Analysis may be adjusted for stage at diagnosis. The effect of attending a screen in a particular 5-year age band (e.g. 60–64), in the pilot phase, or 2-year age band (e.g. 60–61), in the main phase, on incidence from advanced stage primary CRC in the subsequent 5-year age band (i.e. 62–66 or 65–69, respectively) will also be investigated, as performed for the cervical screening audit [17]. The results of this analysis should be very similar to those obtained from the analysis of ‘time since last screen’ after stratifying the analysis by age at diagnosis/pseudo-diagnosis.

In the main phase of the study, the effect of invitation to bowel screening and attendance at screening prior to the *index screen* of the case will also be assessed among screen-detected cases matched to controls screened within the same screening interval (Table 1 Design 5).

## Discussion

This study protocol addresses the central question of the effects of the NHS BSCP in terms of the benefits on incidence of advanced primary colorectal cancer, defined as pathological Duke’s stage B or worse using a retrospective matched case–control study approach.

The attraction of the case–control evaluation strategy resides in that for a study nested within the cohort of individuals offered screening, with screening exposure data prospectively recorded, this design confers a reliability and an interpretability comparable with those of a prospective evaluation, while being quick to perform as it requires no further follow-up. It also directly relates the clinical endpoint to the screening history at an individual level, and it requires a relatively small number of cases and corresponding controls. In addition, the case–control design allows the assessment of what actually happened in the population during service screening, taking into account natural variation. It also has the flexibility to question aspects of the screening regime, for example in relation to intervals and target populations, which were not possible to address using RCT data.

The case–control design is potentially prone to a number of biases, in particular some that could confer a bias *in favour* of screening. However, with careful design and analysis, one can minimize the risk of biased results.

When the programme is mature, new case–control studies will be designed to assess the impact of the programme on mortality from colorectal cancer.

### Ethics

The study protocol was reviewed by the NHS BCSP Evaluation Group and approved by the UK Department of Health. The study protocol was submitted to the Health Research Authority (HRA) NHS Health & Social Care Research & Development Ethics Committee (REC) and to the Confidential Advisory Group (CAG, formerly the National Information Governance Board for Health & Social Care (NIGB) via e-submission through the Integrated Research Application System (IRAS). Ethical approval was obtained from the London City & East National Research Ethics Committee (reference number 14/LO/0826), and permission to process identifiable information of patients in England without consent (under section 251 of the NHS Act 2006) was obtained from the CAG (reference number 14/CAG/1020).

## Abbreviations

CRA: Colorectal adenoma
CRC: Colorectal cancer
FIT: Faecal immunochemical test
gFOBt: Guaiac faecal occult blood test
HSCIC: Health & social care information centre
IMD: Index of multiple deprivation
NCDR: National cancer data repository
NHAIS: National health application & infrastructure services
NHS BCSP: NHS bowel cancer screening programme
NHS BSP: NHS breast screening programme
NHS CSP: NHS cervical screening programme
NYCRIS: Northern and Yorkshire Cancer Registry and Information Service
PCDP: Pre-Clinical screen-Detectable Period
PRU: Policy Research Unit in Cancer Awareness, Screening and Early Diagnosis
RCT: Randomised controlled trial.
